# Supplementary material for: Steppe lemmings and Chinese hamsters as new potential animal models for the study of the Leishmania subgenus Mundinia (Kinetoplastida: Trypanosomatidae)
Source: PLoS Negl Trop Dis. 2024 May 13;18(5):e0011897. doi: 10.1371/journal.pntd.0011897 (PMC11090356; doi:10.1371/journal.pntd.0011897)
Supplement: Attachment — Submitted filename: Responses to Reviewere.pdf [file pntd.0011897.s004.pdf]

We sincerely thank all the Reviewers for their positive evaluation of the manuscript and their insightful comments. We have answered all questions and completed all requested information and believe that this version of the manuscript will meet all the criteria for publication.

A detailed list of responses follows:

Methods

Reviewer #1: (No Response)

Reviewer #2: This study presents the experimental evidence of the long-term survival of Mundinia parasites in new potential animal models as well as their infectiousness to vectors. Thus, the research content is interesting and worth publishing but needs correction and more critical discussion.

Major comments:

1. The authors mentioned *Culicoides* as the most probable vectors of *Mundinia* species with the previous evidence of successful metacyclogenesis and transmission to animal model. In addition, it appears that transmission of human-infecting *Mundinia* species entirely failed with *Phlebotomus argentipes*. However, *Phlebotomus*, not *Culicoides*, was used for xenodiagnosis in the present study. What is the rationale of using *Phlebotomus* for xenodiagnosis in this research? Additionally, xenodiagnosis was not mentioned in the objective. So, the authors should include the rationale and objective regarding this in the introduction.

There is no proven natural vector of *Mundinia* and *Culicoides sonorensis* colony is available only in the Pirbright institute, UK. Transporting biting midges for each xenodiagnostic trial would be logistically and financially very challenging, so we decided to use a colony of sand flies available in our insectary. *Phlebotomus duboscqi* feed well on rodents and our colony is large enough to allow such experiments. From a methodological point of view, it was possible to use sand flies because the first part of *Leishmania* development in sand flies is non-specific and parasites survive for several days in digested blood inside of peritrophic matrix even in refractory vectors (doi: 10.1603/ME12053, doi: 10.1186/1756-3305-7-222, doi: 10.1186/1756-3305-6-186). Engorged *P. duboscqi* females were left alive for two days before DNA isolation and PCR analysis to allow parasites to multiply. Xenodiagnoses trials were mentioned in the Introduction (line 92) and the reason why *P. dubosqci* was used for xenodiagnoses was added to the Methods (lines 125-128), because we believe, these methodological details fit there better compared to introduction section.

2. Line 80: Only four cases of *L. orientalis* has formally been reported. Please include two more relevant references as follows:
doi: 10.1186/s13071-018-2908-3 and doi: 10.4269/ajtmh.22-0385.

References were included in the manuscript.

3. Line 90: What are "a sink" and "a source"? To facilitate the general readers, please clarify these two terms.

Sentence was changed to: However, it should be noted that detection of parasites or their DNA does not imply the reservoir potential of the host, since parasites can be present in the organism

incidentally and be detectable, but not transmittable to other hosts. These organisms are so called "sinks" of infection. The opposite are so called "sources", where parasites are detectable and further transmittable to other hosts (28). – We believe, that the general readers will now fully understand the meaning of the "sink and sources" hypothesis. If not, we believe, that primary citation, which is included at the end of the sentence, will suffice.

4. Figure 2 shows only the anatomical distribution of parasites in steppe lemmings. Do the authors have the similar figure for those in Chinese hamsters?

The figure was added to Chapter 3.2. as Figure 1.

5. The authors proved the organ infection by only nested-PCR. Do the authors have the histological validation?

Histological analyses were not performed, and whole organs were harvested for molecular analysis by PCR and qPCR, as these experiments were pioneering and the results serve as evidence of the susceptibility of Chinese hamsters and Steppe lemmings and the resistance of BALB/c mice to *Mundinia* after intradermal inoculation. More detailed tissue analyses are planned in future research.

6. Lines 372-374: How do the sand fly bites influence the course of infection? Please describe more.

Information on this topic was completed and is now provided on lines 376-380 in the following version: … consistent with previously stated hypothesis of enhancing effect of sand fly bites to the course of infection by Vojtkova et al., who observed significantly faster and to a larger size lesion development in *L. major* infected BALB/c mice exposed to repeated bites of *P. dubosqci* compared to the unexposed group of mice, and the tissue parasite load was higher in rodents exposed to repeated bites (55). Also in hamster model, increased transmissibility of *L. donovani* was observed following multiple exposures of hamsters to *Lutzomyia longipalpis* bites (56).

(55) Vojtkova, B., Frynta, D., Spitzova, T., Lestinova, T., Votypka, J., Volf, P., & Sadlova, J. (2021). Repeated sand fly bites of infected BALB/c mice enhance the development of Leishmania lesions. *Frontiers in Tropical Diseases*, *2*, 745104.

(56) Valverde, J. G., Paun, A., Inbar, E., Romano, A., Lewis, M., Ghosh, K., & Sacks, D. (2017). Increased transmissibility of Leishmania donovani from the mammalian host to vector sand flies after multiple exposures to sand fly bites. *The Journal of Infectious Diseases*, *215*(8), 1285-1293.7.

Lines 375-376: As recorded, *L. chancei* (previously known *L.* sp. Ghana) has been known to cause cutaneous leishmaniasis in Ghana. Visceral leishmaniasis has never been epidemiologically reported before for this species. Thus, this finding of cutaneous and visceral symptoms in this study unveil the possible risks of visceral involvement in humans by this species. I suggest the authors to include this issue in this discussion.

Thank you for this note. We added text to the lines 401-405.

The visceralisation observed in steppe lemmings is unparalleled to human infections described in Ghana and neighbouring countries. Here, among 8000 cases of leishmaniasis none of the cases was described as visceral, only skin symptoms were observed (Kwakye-Nuako et al. 2015). However, the results of current study suggest that *L. chancei* could have the potential to visceralize, for example, in immunocompromised individuals such as HIV-positive people.

8. Lines 377-388: Surprisingly, *L. orientalis* displayed no cutaneous lesions but caused visceral symptoms resulting in deaths in steppe lemmings. Conversely, in humans this *Mundinia* species has previously been known to be mainly responsible for cutaneous leishmaniasis. Please discuss more.

We have added a discussion on this topic to the lines 406-409*: Leishmania chancei* is most closely related to *L. orientalis* from Thailand (Anugulruengkitt et al. 2023), where variable human disease manifestations correlated with patients' immunocompetence status can be observed. The type strain LSCM4 (Jariyapan et al. 2018) used also in our study and the strain CULE5 (Anugulruengkitt et al. 2023) caused simple skin lesions in immunocompetent patients while in AIDS patients, the strain PCM2 Trang and the anonymous isolate from Kanchanaburi caused disseminated cutaneous leishmaniasis and visceral disease (Supsrisunjai et al 2017, Bualert et al. 2012).

9. What is the limitations and implications/implementation (in terms of diagnostics and therapeutic development) in this study? The authors should critically discuss more.

In general, wild rodents represent a novel approach to the study of host competence and host-parasite relationships (Loria-Cervera and Andrade-Narvaez 2014). The use of only one inbred genotype may not correspond to the natural variability in parasite's tissue tropism, the dynamics and duration of infection or the infectiousness to vectors. Since the manifestations of human diseases caused by *Mundinia* are variable and show almost as polymorphic disease course as those caused by *Leishmania* and *Viannia* subspecies, it would be optimal to introduce a wider range of model animals. Conversely, for studies aimed at drug testing or the development of diagnostic methods, it would be preferable to use less genetically variable models, either obtained by long-term inbreeding of these rodent species or by searching among existing inbred mouse models different from BALB/c. The results of this work must be considered pioneering and indicative of the direction of future research.

We added this text to lines 430-437.

Minor comments:
1. Lines 73-75: Please recheck the format of the reference.

Done

2. The term "leishmania" which is a genus name need to be italic and start with the capital letter "L". Please correct throughout the manuscript.

Done

Reviewer #3: The hypothesis and objectives of the study were clearly stated and the design of study was appropriate. To fulfil requirements for animal model statistics and ethics, the number of animals used which were a group of 10 animals for infection of each species was appropriate and suitable. Fisher's exact test was used for statistical analysis to compare infection rates which is

suitable for animal infection model studies. Ethics approval numbers have been documented in the manuscript to prove ethical and regulatory requirements have been met.

Results

Reviewer #1: (No Response)

Reviewer #2: -

Reviewer #3: The objective of these analyses is to test new animal models for leishmania infection studies. The analysis followed as according to the study plan. A total of five species and 4 strains of one of the species, Leishmania martiniquensis were tested systemically in 3 rodent species, mice (BALB/c strain), steppe lemmings and Chinese hamsters. Results from the infections in the 3 infection animal models were presented logically with a comprehendible narrative. Negative infection rates were first presented for the BALB/c mice model, followed by asymptomatic infection demonstrated with the Chinese hamster model and symptomatic infection with the steppe lemming model. Comprehensive details in observations and analysis were included as part of the results which provided an in depth insight that is useful for future work using these animal models for Leishmania infection studies. Figures and tables summarise the data accurately and convey the message effectively for the purpose of these studies.

Conclusions

Reviewer #1: (No Response)

Reviewer #2: -

Reviewer #3: The conclusions are supported by the data presented. Steppe lemmings and Chinese hamsters were demonstrated to be suitable animal models for Leishmania infection studies particularly for the Mundinia species while BALB/c mice were unsuitable as they were resistant to infection from these parasite species. Limitations for these analyses were mentioned in the discussion and suggested for further work that could be established based on results from these studies.

Editorial and Data Presentation Modifications?

Reviewer #1: (No Response)

Reviewer #2: -

Reviewer #3: Line 216: (maximum 2,4x104) - check again if it is supposed to be 2,4 or 2.4, or 2-4?

Checked and repaired, correctly it is 2.4×10^4

Line 254: space between 88.9 and % symbol should be removed.

Space was removed

Summary and General Comments
Use this section to provide overall comments, discuss strengths/weaknesses of the study, novelty, significance, general execution and scholarship. You may also include additional comments for the author, including concerns about dual publication, research ethics, or publication ethics. If requesting major revision, please articulate the new experiments that are needed.

Reviewer #1: The manuscript titled " Steppe lemmings and Chinese hamsters as new potential animal models for the study of the leishmania subgenus Mundinia (Kinetoplastida: Trypanosomatidae)" has been reviewed. The authors have effectively highlighted the potential of these species to serve as reservoir hosts for Mundinia. The manuscript holds value and contributes to the scientific community by introducing two new potential laboratory animal model species, Steppe Lemmings and Chinese Hamsters, for studying the leishmania subgenus Mundinia. However, the authors need to add the following queries for improvements.

-Line 73,75,79: replacing "Leishmania" with "L." and apply this change consistently throughout the manuscript.

Done

-Line 119: Why did the authors choose to inject promastigotes into the left ear pinnae? Is this practice common in the literature or influenced by prevalence? Please discuss and consider including relevant references.

The ear pinna was chosen because it is the most appropriate site for monitoring the development of lesions and it is also the site where sand flies naturally bite, therefore suitable for xenodiagnoses. We added this sentence along with a reference to literary sources to the text on the lines 119-121.

-Line 121 states that xenodiagnoses were performed at weeks 5, 10, 15, and 20 post-infection using Phlebotomus duboscqi. Why did the authors choose Ph. duboscqi for this study?

This answer is the same as for the Reviewer 2: There is no proven natural vector of *Mundinia* and *Culicoides sonorensis* colony is available only in the Pirbright institute, UK. Transporting biting midges for each xenodiagnostic trial would be logistically and financially very challenging, so we decided to

use a colony of sand flies available in our insectary. *Phlebotomus duboscqi* feed well on rodents and our colony is large enough to allow such experiments. From a methodological point of view, it was possible to use sand flies because the first part of *Leishmania* development in sand flies is non-specific and parasites survive for several days in digested blood inside of peritrophic matrix even in refractory vectors (doi: 10.1603/ME12053, doi: 10.1186/1756-3305-7-222, doi: 10.1186/1756-3305-6-186). Engorged *P. duboscqi* females were left alive for two days before DNA isolation and PCR analysis to allow parasites to multiply. This information was added to the Methods (lines: 123-129).

Additionally, did the authors collect blood from the animals weekly for detecting Leishmania? Please provide a discussion on this matter.

We did not collect blood because leishmania are very rare in rodent blood samples (at least using the natural method of infection by inoculation into the pinna). Even visceral *L. donovani* was present in BALB/c mice mainly in the skin and lymph nodes in addition to the spleen and liver, blood was positive in only 1/10 animals (Sadlová et al. 2015). In addition, blood sampling is more invasive in steppe lemmings and Chinese hamsters than in laboratory mice (while mice can easily have blood drawn from the tip of their tail and it is not too stressful for them, these rodent species have a short tail unsuitable for regular blood sampling).

-Line 171,181,206: Clarify the number of animals. Why are the conditions not consistent? Is there duplication in testing?

It was a typo, the groups always consisted of 10 animals. The typo was corrected.

-Line 216: Replace 'maximum 2,4x10^4' with '2.4 x 10^4'."

Replaced

Reviewer #2: The revisions of the manuscript are needed as mentioned above.

Reviewer #3: Overall, this manuscript is well written, easy to read and comprehend. The introduction provided sufficient insights into the topic about Leishmania as a pathogen and its significance to human health. The methods are written clearly with sufficient detail for replication of results. The study is novel and provides additional knowledge to the field, enabling scientists to further investigate infection related studies for Leishmania Mundinia subspecies, potentially enabling researchers to study new therapeutics for this disease. I would like to question:

Regarding the rationale for the selection of animals for xenodiagnoses trials, it is only indicated for the BALB/c mice and steppe lemmings infection studies that 4 animals per group were selected for xenodiagnoses trial. However, it is not indicated in the section 3.2 Development of Mundinia in Chinese hamsters about the number of animals that had been selected for the xenodiagnoses trial. 1) How many animals were chosen for each Leishmania species infection for the Chinese hamster xenodiagnoses trial? - to indicate this in the manuscript within the section.

The animals were 4 per group also in Chinese hamsters. The information was added to lines 192-193.

2) Is there a reason why these 4 animals out of 10 animals in each group were selected? - to explain the reason behind how these animals were selected in the manuscript.

Specimen for xenodiagnoses were chosen randomly at the beginning of the experiment and the same animals were used for all xenodiagnoses trials.

This sentence was added to the lines 130-131.
